# p53 is required for nuclear but not mitochondrial DNA damage-induced degeneration

**DOI:** 10.1038/s41419-020-03373-1

**Published:** 2021-01-20

**Authors:** Matthew J. Geden, Selena E. Romero, Mohanish Deshmukh

**Affiliations:** 1 Department of Cell Biology and Physiology, University of North Carolina, Chapel Hill, NC 27599 USA; 2 Neuroscience Center, University of North Carolina, Chapel Hill, NC 27599 USA

**Keywords:** Cell biology, Neuroscience

## Abstract

While the consequences of nuclear DNA damage have been well studied, the exact consequences of acute and selective mitochondrial DNA (mtDNA) damage are less understood. DNA damaging chemotherapeutic drugs are known to activate p53-dependent apoptosis in response to sustained nuclear DNA damage. While it is recognized that whole-cell exposure to these drugs also damages mtDNA, the specific contribution of mtDNA damage to cellular degeneration is less clear. To examine this, we induced selective mtDNA damage in neuronal axons using microfluidic chambers that allow for the spatial and fluidic isolation of neuronal cell bodies (containing nucleus and mitochondria) from the axons (containing mitochondria). Exposure of the DNA damaging drug cisplatin selectively to only the axons induced mtDNA damage in axonal mitochondria, without nuclear damage. We found that this resulted in the selective degeneration of only the targeted axons that were exposed to DNA damage, where ROS was induced but mitochondria were not permeabilized. mtDNA damage-induced axon degeneration was not mediated by any of the three known axon degeneration pathways: apoptosis, axon pruning, and Wallerian degeneration, as Bax-deficiency, or Casp3-deficiency, or Sarm1-deficiency failed to protect the degenerating axons. Strikingly, p53, which is essential for degeneration after nuclear DNA damage, was also not required for degeneration induced with mtDNA damage. This was most evident when the p53-deficient neurons were globally exposed to cisplatin. While the cell bodies of p53-deficient neurons were protected from degeneration in this context, the axons farthest from the cell bodies still underwent degeneration. These results highlight how whole cell exposure to DNA damage activates two pathways of degeneration; a faster, p53-dependent apoptotic degeneration that is triggered in the cell bodies with nuclear DNA damage, and a slower, p53-independent degeneration that is induced with mtDNA damage.

## Introduction

The maintenance of cellular DNA integrity and the elimination of cells that have sustained excessive DNA damage is fundamentally important for all organisms. While mitochondrial DNA (mtDNA) is several orders of magnitude smaller than the nuclear genome^1^, maintenance of mtDNA integrity is crucial as mutations in mtDNA have been shown to be important in a number of diseases including cancer, neurodegenerative diseases, and aging^2,3^.

Chemotherapeutic drugs that induce DNA damage are frontline treatments for cancer therapy^4^. These DNA-damaging events engage a number of downstream pathways depending on the degree of damage. While low levels of DNA damage can be repaired, induce cell cycle arrest, senescence, or differentiation, excessive damage can lead to cell death via apoptosis^5^. Specifically, nuclear DNA damage activates the classical intrinsic pathway of apoptosis that is initiated by p53, mediated by the proapoptotic protein Bax, and executed by caspases such as Caspase-3 (Casp3)^6,7^. Recently, it has become increasingly evident that exposure of cells to genotoxic drugs affects not just nuclear DNA but also mitochondrial DNA^8^. However, because it is challenging to separate the distinct consequences of mtDNA insult from nuclear DNA insult, it is unknown whether mtDNA damage activates the same cellular response as nuclear DNA damage.

The majority of mtDNA damage, especially oxidative damage by reactive oxygen species that are generated as a byproduct of oxidative phosphorylation^9^, is detected and rapidly repaired by the highly effective base excision repair (BER) pathway^1,10,11^. While other DNA repair pathways have been reported in the mitochondria, such as single-strand and mismatch repair^10,11^, it is unclear how much these pathways functionally contribute to mtDNA repair. Notably however, mitochondria appear to completely lack the nucleotide excision repair (NER) pathway, which is typically used to repair nuclear DNA insults from chemotherapeutic drugs such as cisplatin-induced intra-strand crosslinks^1,10,11^.

In contrast to our knowledge of the pathways important for mtDNA repair and during biogenesis, the pathways activated by sustained and selective mtDNA damage remain virtually unknown. Several studies have utilized the “mutator” mice, which carry a knock-in inactivating mutation in the exonuclease domain of the mitochondrial DNA polymerase POLG, to examine the outcome of mtDNA damage. As this mutant POLG lacks its proof-reading activity, the mutator mice gradually accumulate persistent mtDNA mutations and exhibit a premature aging phenotype^12,13^. A second mouse model of mtDNA damage is one where mtDNA double-stranded breaks can be induced by the expression of a mitochondrially-targeted restriction endonuclease (Pst1)^14,15^. Even a transient induction of mtDNA double-stranded breaks is sufficient to induce an aging phenotype in these mice^16,17^. Both these mouse models underscore the importance of maintaining mtDNA fidelity and show that chronic accumulation of mtDNA mutations cause premature aging. In addition to mouse models, a few studies have also induced mtDNA damage in cell lines. Depletion of the exonuclease EXOG, which resulted in persistent single stranded brakes in mtDNA (but not nuclear DNA), induced cell death^18^. Interestingly, exposure of cancer cell lines to chemotherapeutic drugs that are modified to target the mitochondria can also induce cell death with markers of apoptosis^19–21^. However, whether the apoptotic pathway mediates mtDNA damage-induced cell death is not known.

To induce selective damage to mtDNA but not nuclear DNA, we have taken advantage of the unique biology of neurons where the cell body and axons are spatially distinct. While neuronal cell bodies (soma) contain both nuclear DNA and mtDNA, the only source of DNA in axons is mtDNA from the axonal mitochondria. Importantly, in order to selectively expose only the axonal mitochondria to DNA damage, we utilized compartmentalized microfluidic chambers. Our results show that exposure of axons to the DNA damaging drug cisplatin is sufficient to induce mtDNA damage which resulted in the selective degeneration of the exposed axons. Here, we examine the critical importance of the p53-depedent apoptotic pathway in mediating the mtDNA damage-induced degeneration in neurons.

## Results and discussion

### Microfluidic chambers permit the selective induction of mtDNA damage

**Fig. 1:**
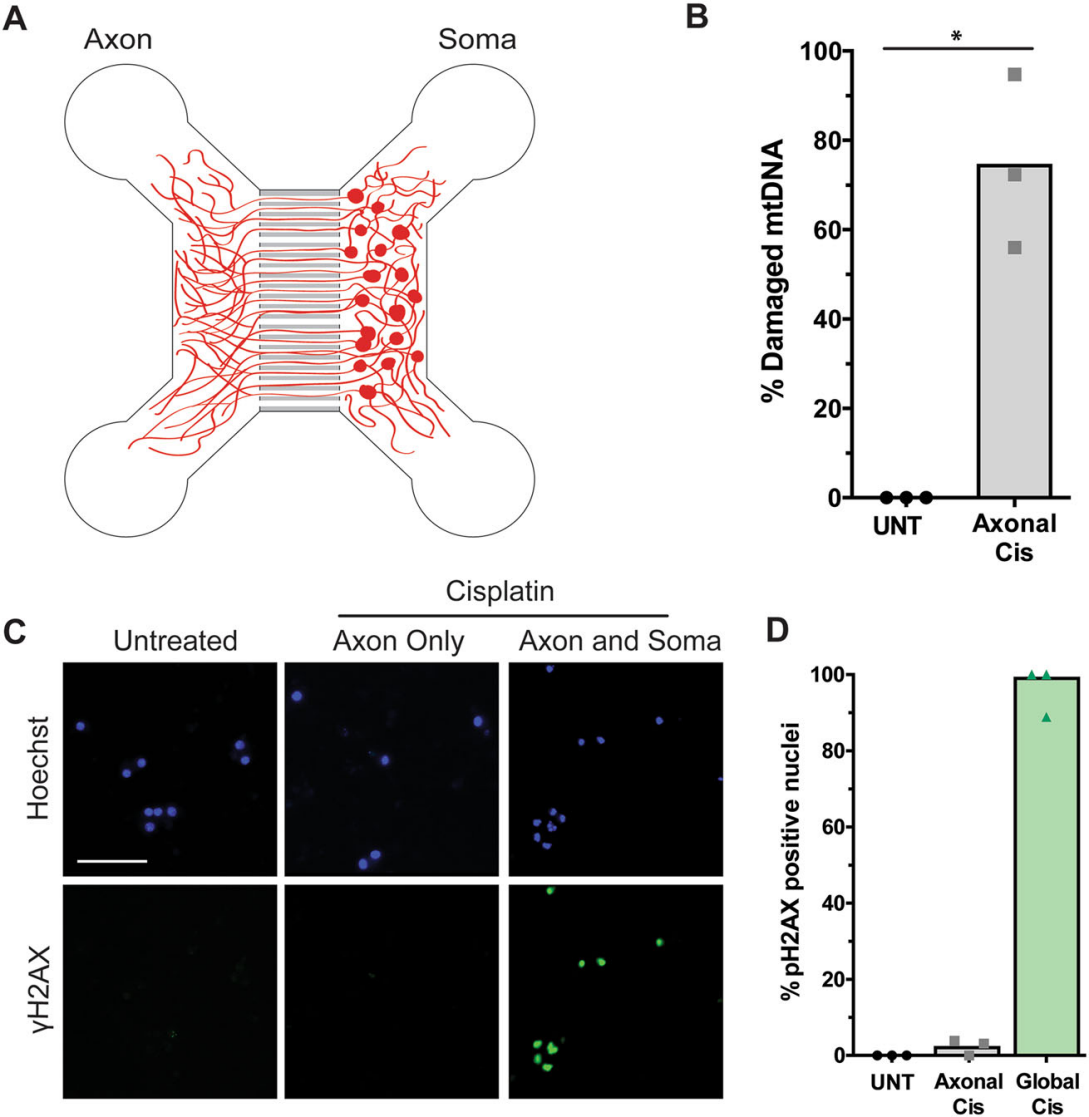
Axon-only exposure to cisplatin in compartmentalized cultures induces axonal mtDNA damage without damaging nuclear DNA. **A** Illustration of the microfluidic chambers where neurons are placed in the soma compartment and allowed to extend their axons into the axon compartment through the microgrooves separating the two compartments. **B** Neurons in microfluidic chambers were either left untreated (UNT) or treated with 20 µM cisplatin to the axon chamber (Axonal Cis). Shown is quantification of mtDNA damage represented as % undamaged mtDNA in axonal mitochondria after 24 h of exposure as compared to untreated controls (*n* = 3); individual data points and mean are depicted. Paired *t*-test (two-tailed) (*p < 0.05). **C** Neurons in microfluidic chambers were left untreated (UNT) or treated with cisplatin to only the axon chamber (Axon only) or treated with cisplatin to both the compartments (Axon and Soma; Global). Shown are images of the neuronal soma stained with antibodies to ɣH2AX and the corresponding images of nuclei stained with Hoechst at 120 h after treatment with 20 µM cisplatin. While ɣH2AX foci in the nucleus are observed in globally treated neurons (cisplatin added to both axon and soma compartments), virtually no ɣH2AX foci are visible upon cisplatin exposure to the axon-only compartment. The scale bar illustrated here represents 100 μm. **D** Quantification of ɣH2AX staining (*n* = 3), individual data points and mean are depicted.

To induce selective damage to mtDNA but not genomic DNA, we used primary mouse neurons in microfluidic-based compartmentalized chambers (Fig. 1A). These microfluidic chamber devices have two compartments, “soma” and “axon”, separated by a series of microfluidic grooves. The soma and axon compartments maintain strict fluidic separation, which has been rigorously demonstrated in previous studies^22,23^. In these chambers, neurons are seeded into the soma compartment and over several days extend axons through the microfluidic grooves into the axon compartment. While the soma compartment contains nuclear DNA, as well as mtDNA (from both the soma and proximal axons), the axon compartment contains only mtDNA. Thus, these chambers allow for the exposure of only the axon chamber to DNA damaging agents to selectively damage axonal mtDNA without affecting the nuclear DNA contained in the cell bodies in the soma compartment.

Exposure of DNA damaging drugs, such as cisplatin, to mass cultures of cells is known to induce damage to both nuclear and mitochondrial DNA^24^. Importantly, cisplatin induces intrastrand crosslinks that are not predicted to be repaired in mitochondria, as mitochondria lack NER^1,10,11^. Thus, we examined whether the directed exposure of cisplatin to only the axon compartment in our neuronal microfluidic model was sufficient to induce mtDNA damage. Using an established PCR amplification assay for detecting mtDNA damage^25,26^, we found that axonal cisplatin exposure induced substantial damage to mtDNA in the axon compartment (Fig. 1B). Importantly, we examined the cell bodies for γH2AX staining, a marker of nuclear DNA damage^27^, to determine whether the addition of cisplatin to only the axon chamber induced any nuclear DNA damage. γH2AX staining was virtually undetectable in the nuclei when cisplatin was added only to the axon chamber (Fig. 1C, D). In contrast, and as expected, robust γH2AX staining was observed in the nuclei when cisplatin was added to both the soma and axon compartments (Fig. 1C, D). Together, these results show that addition of cisplatin to the axon chamber induced mtDNA damage to the targeted axons, without inducing nuclear DNA damage.

### Axonal mtDNA damage is sufficient to trigger axon degeneration

**Fig. 2:**
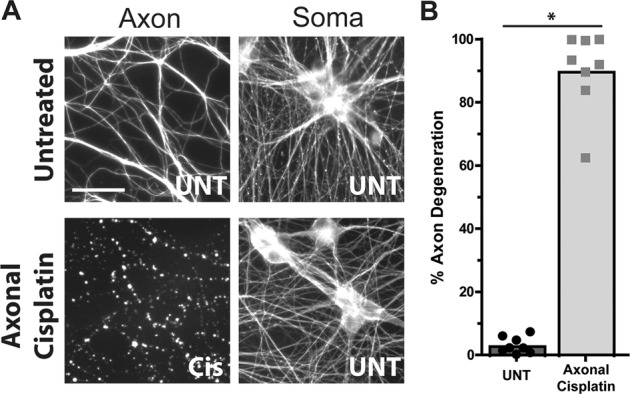
Cisplatin addition to axons is sufficient to induce localized axon degeneration. **A** Neurons in microfluidic chambers were left untreated (UNT; Top panels) or had their axons alone (and not their soma/nucleus) exposed to cisplatin (Axonal Cisplatin; 20 μM; Bottom panels) for 120 h and stained for tubulin. The scale bar represents 20 μm. Representative images are shown from *n* = 3. **B** Quantification of the axon degeneration from these experiments is shown on the right (*n* = 8), individual data points and mean are depicted. Paired *t*-test (two-tailed) (*p < 0.05).

The majority of studies examining the outcome of DNA damage have examined the outcome after the entire cell, both nuclear DNA and mtDNA, were exposed to damage. In these contexts, sustained DNA damage is well known to induce robust degeneration as a result of apoptotic cell death^7^. To determine if sustained mtDNA damage, in the absence of nuclear DNA damage, was also capable of inducing degeneration we added cisplatin to only the axon compartment and, strikingly, found that cisplatin induced robust degeneration of the exposed axons (Fig. 2A, B). Interestingly, we also observed that the cell bodies and proximal axons in the soma compartment remained intact, which is consistent with our findings that axonal cisplatin exposure did not induce DNA damage in the soma compartment. In axons treated with cisplatin we observed greater than 90% axon degeneration after 5 days of cisplatin exposure, while untreated axons remained healthy with minimal axon degeneration (Fig. 2).

**Fig. 3:**
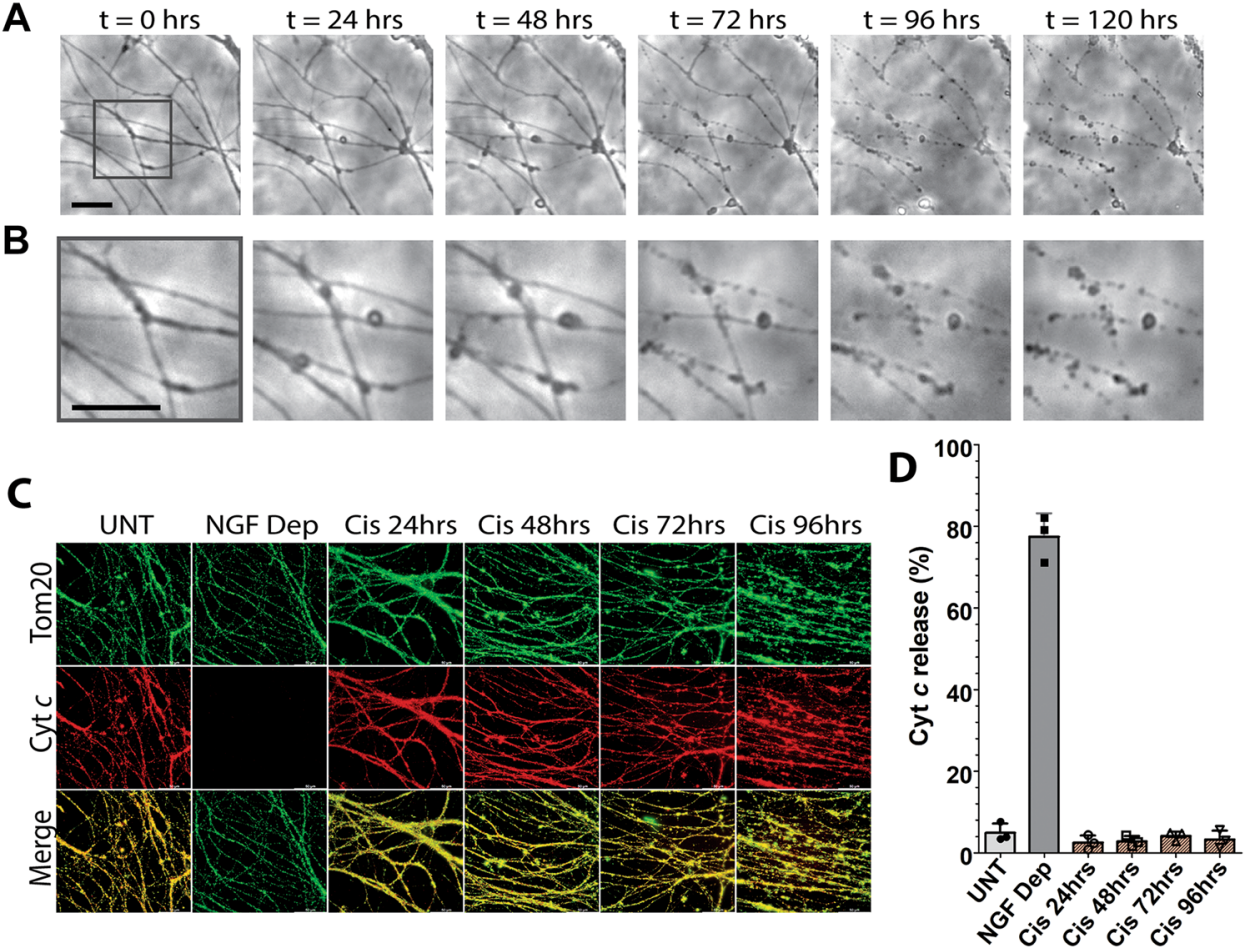
Axonal mtDNA damage induces complete axon degeneration after 96 h of cisplatin exposure without the release of cyt *c*. Neurons were cultured in microfluidic chambers and had only their axons exposed to cisplatin (20 μM) at *t* = 0 h and were monitored for degeneration by microscopy every 24 h for 120 h total. **A** Depicted in the top row are phase contrast representative images of axons in chambers illustrating how the same axons degenerate over 120 h, **B** Zoomed-in region from **A** that demonstrates the finer signs of damage that precede complete degeneration. The black scale bar for both rows is 20 μm. **C** Immunostaining of cyt *c* and TOM20 (mitochondrial marker) to assess release of cyt *c* from axonal mitochondria treated with cisplatin. NGF deprivation of neurons for 48 h (in the presence of QVD-OPH) is included as a positive control for cyt *c* release. The quantification of cyt c release is shown in **D**, individual data points and mean are depicted.

The kinetics of axon degeneration in sympathetic neurons in other contexts has been well defined, with degeneration being observed 8 h after axotomy^28^, 24–48 h after apoptosis^29^, and 48–72 h after axon pruning^30^. To examine the timecourse of axon degeneration after mtDNA damage, we exposed axons to cisplatin in microfluidic chambers and monitored them every 24 h over the course of degeneration. Interestingly, we found axon degeneration after mtDNA damage to occur much more slowly than other contexts of axon degeneration. Axons began to show signs of membrane blebbing by 24 h after cisplatin exposure (Fig. 3A, B). The size and number of these blebs continued to increase over time until 96 h at which point the axons began to degenerate with complete degeneration occurring by 120 h (Fig. 3A, B).

Next, we examined whether damage to axonal mtDNA resulted in the elevation of reactive oxygen species (ROS) in axons. We conducted a time course study and found that ROS levels were markedly elevated in the axons by 24 h of cisplatin exposure and remained high until overt axonal degeneration (Supplemental Fig. S1A). A key event during apoptosis is the permeabilization of mitochondrial outer membrane and the release of cytochrome *c* (cyt *c*). Thus, we also examined the status of cyt *c* in the mitochondria of cisplatin-treated axons. Our results show that mtDNA damage did not trigger the release of cyt *c* from the mitochondria at any time during cisplatin-induced axon degeneration (Fig. 3C, D). Consistent with the lack of mitochondrial permeabilization, we found that mitochondrial membrane potential was also maintained in the mitochondria of cisplatin-treated axons (Supplemental Fig. S1B).

### mtDNA damage-induced axon degeneration is not dependent on the p53 DNA damage response pathway

A key protein activated in response to genomic DNA damage is p53. In cases of minor DNA damage, p53 can arrest cell cycle and initiate DNA repair pathways. However, in situations of sustained and significant DNA damage, p53 can instead trigger cell death via activation of the apoptotic pathway^31^. The importance of p53 in this context is underscored by the fact that cells, including neurons, deficient in p53 are protected from DNA damage-induced cell death^32–37^.

**Fig. 4:**
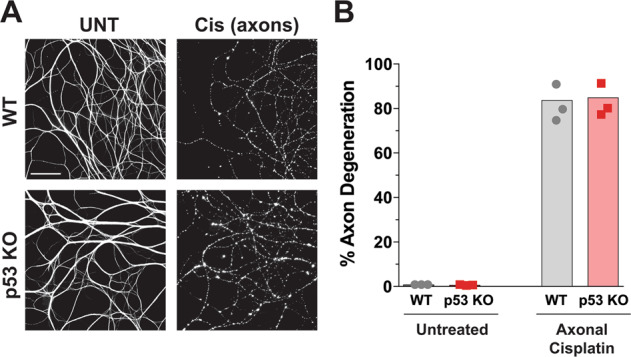
p53-deficiency failed to protect axons from cisplatin-induced mtDNA damage. **A** Neurons were isolated from wildtype and p53-deficient mice and treated with cisplatin exposed onto to the axon compartment (Axonal Cisplatin; 20 μM) for 120 h and stained for tubulin (*n* = 3). The scale bar depicts 100 μm. **B** Quantification of the axon degeneration of the experiments in **A** (*n* = 3), individual data points and mean are depicted.

Interestingly, p53 is known to translocate to the mitochondria in response to DNA damage^38,39^ and has been implicated in the repair and biogenesis of mtDNA^40–42^. However, whether p53 can also mediate cell degeneration in response to mtDNA damage is not known. To determine the role of p53 in regulating mtDNA damage induced degeneration, we examined the requirement of p53 in our model of axonal cisplatin exposure. Specifically, neurons from p53-deficient mice were plated into the microfluidic chambers and cisplatin was added selectively to the axon compartment. Interestingly, p53-deficient axons were not protected from degeneration induced by axonal exposure to cisplatin (Fig. 4A) as both the p53-deficient and wildtype neurons underwent similar degrees of degeneration (Fig. 4B). Given the crucial role of p53 in the induction of apoptosis in response to genomic DNA damage, these results highlight the differential requirement of p53 in nuclear versus mtDNA damage.

### mtDNA damage-induced axon degeneration is not mediated by the pathways of apoptosis, axon pruning, or Wallerian degeneration

**Fig. 5:**
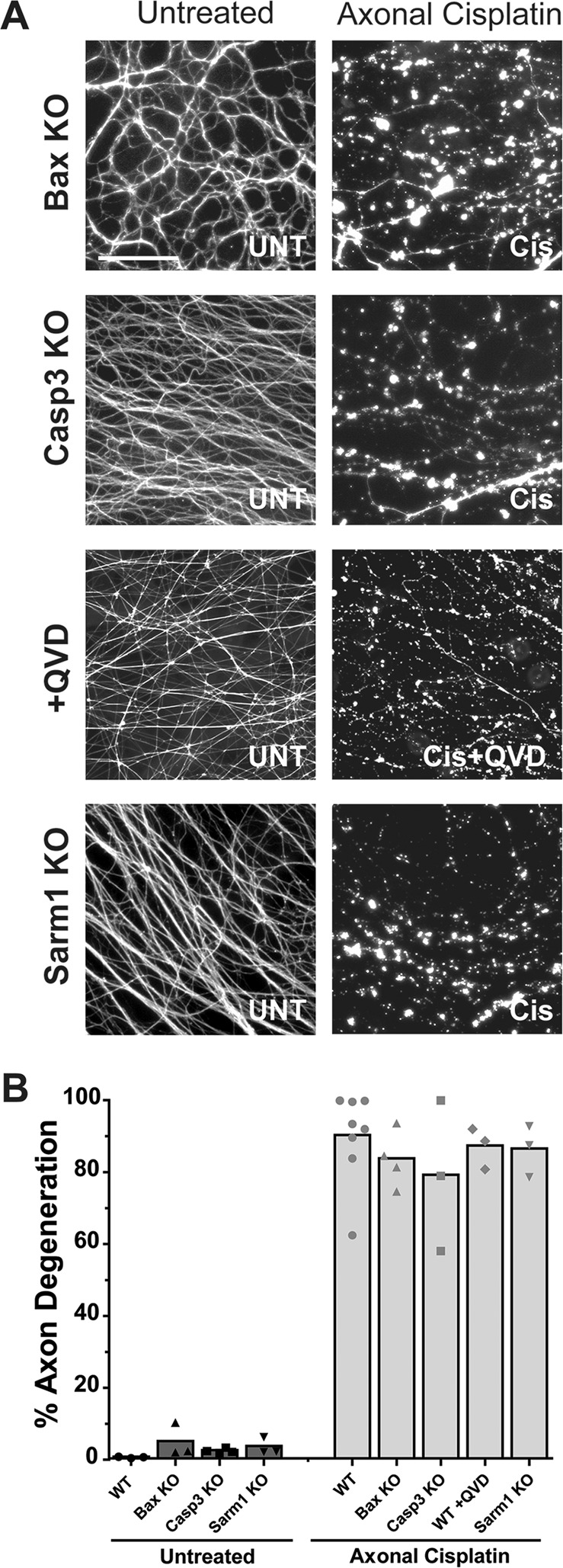
Cisplatin-induced axon degeneration is not mediated by the known pathways of axon degeneration. **A** Neurons were isolated from mice, either wildtype or those deficient in Bax, Casp3, and Sarm1 and cultured in microfluidic chambers and either left untreated (UNT) or treated with cisplatin applied only to the axon chamber (Axonal Cisplatin; 20 µM) for 120 h and stained for tubulin. For the +QVD condition, wildtype neurons were cultured as described and treated with cisplatin applied only to the axon chamber in the presence of the pan-caspase inhibitor QVD (20 μM Cis + 25 μM QVD) for 120 h and stained for tubulin. The scale bar here depicts 100 μm**. B** Quantification of the axon degeneration described in **A** (*n* ≥ 3), individual data points and mean are depicted.

As we found axon degeneration induced by cisplatin exposure to be a p53-independent process, we next sought to identify the degenerative pathway activated with selective mtDNA damage. We focused first on the apoptotic pathway as this is the primary mechanism by which cells degenerate in response to DNA damage. Two essential mediators of apoptosis in neurons are Bax and Casp3, as neurons deficient in either Bax or Casp3 are protected from undergoing apoptosis^32,43,44^. Thus, we examined whether Bax and Casp3 were required for mtDNA damage induced axon degeneration. Neurons were isolated from Bax-deficient or Casp3-deficient mice and evaluated for their ability to undergo axon degeneration in response to axonal exposure to cisplatin. Surprisingly, neither Bax-deficient nor Casp3-deficient neurons exhibited any protection against cisplatin-induced axon degeneration (Fig. 5A, B; Supplemental Fig. S2A, B).

Neurons can also engage a specialized pathway of axon pruning, that allows for the selective degeneration of targeted axons, while the cell bodies survive^45^. This selective degeneration of the targeted axons but not of the cell bodies resembles the selective degeneration of axons that we observe in response to axonal cisplatin treatment. While axon pruning, like apoptosis, also involves activation of caspases, it is reliant on the activity of an additional caspase, Caspase-6 (Casp6). To determine if axon pruning, or any other caspase-dependent pathway, were involved in mtDNA damage induced axon degeneration we used the pan-caspase inhibitor Q-VD-Oph (QVD). We found that QVD was unable to protect axons in response to axonal cisplatin exposure (Fig. 5C). These data indicate that mtDNA insult-induced axon degeneration is not mediated through axon pruning, nor through any other caspase-mediated degenerative pathway.

Interestingly, neurons can initiate a caspase-independent pathway for the selective degeneration of axons, known as Wallerian degeneration. Wallerian degeneration is activated in the context of axotomy, where damaged axon segments are selectively eliminated^46^. Here, the underlying mechanism of axon degeneration is via catastrophic metabolic failure induced by the activation of Sarm1, an essential mediator of Wallerian degeneration^47^. As sustained mtDNA damage would be expected to induce metabolic failure over time, we examined if the selective axonal degeneration induced by axonal cisplatin exposure was mediated by Wallerian degeneration. To test this, axons of neurons isolated from Sarm1-deficient mice were exposed to cisplatin. We found that Sarm1-deficient neurons were not protected and underwent degeneration similarly to wildtype neurons in response to axonal cisplatin exposure (Fig. 5D; Supplemental Fig. S2C). Thus, neither apoptosis nor the two other well-recognized pathways of axon degeneration appear to be key mediators of mtDNA damage-induced axon degeneration.

### p53-deficiency protects cell bodies and proximal axons from whole-cell cisplatin exposure but fails to protect distal axons

Our finding that deficiency of p53 failed to prevent mtDNA damage-induced axon degeneration was surprising, as p53 deficiency has been reported to protect neurons from degeneration in response to DNA damage^32–37^. However, these previous studies have predominantly utilized markers of degeneration that focus on the neuronal nuclei and cell bodies (e.g., pyknotic nuclei, Propidium Iodide staining, phase bright cell bodies). Thus, in the previous contexts where the mass cultures of p53-deficient neurons (cell bodies and axons) were exposed to DNA damage, the status of axons had not been specifically reported. To reconcile these previous reports on p53 and our current results with directed mtDNA damage to axons, we considered two possibilities of p53 function in somas versus axons. First, that p53 is selectively important for DNA damage-induced degeneration in somas but not in axons. If so, we would predict that p53-deficient neurons, when exposed to global DNA damage (both soma and axons), would protect the soma but result in the complete degeneration of the axons. A second possibility is that exposure of entire neurons to DNA damage activates a faster, nuclear DNA damage-induced p53-dependent apoptotic degeneration while also engaging a slower, mtDNA damage-induced degeneration that is p53-independent. If so, we would predict that p53-deficient neurons exposed to DNA damage would be protected, but only transiently, and that the degeneration would occur without a distinct soma versus axon separation.

**Fig. 6:**
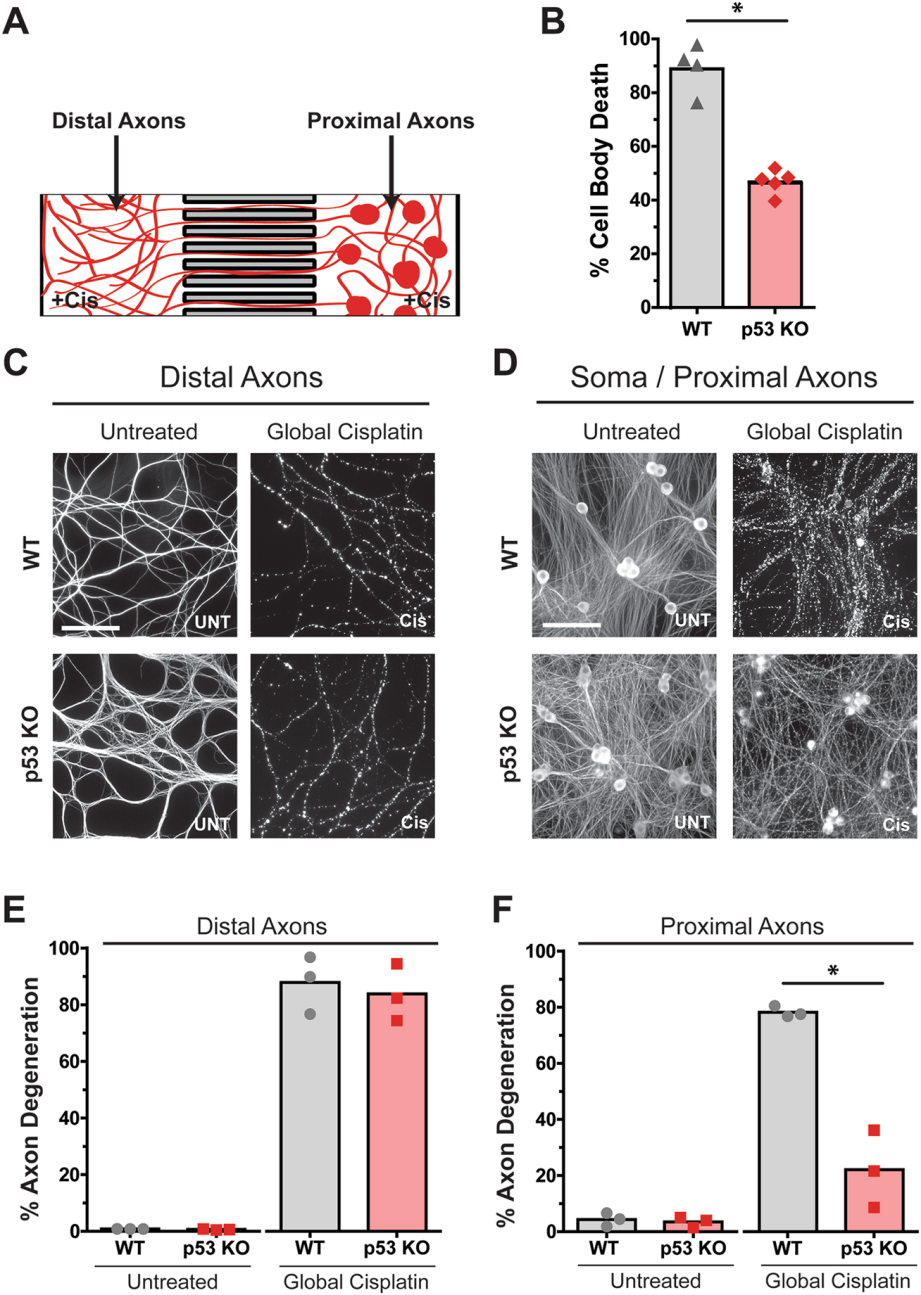
p53 deficiency protects against soma loss from apoptosis but does not protect distal axons from mtDNA induced degeneration with whole cell cisplatin exposure. **A** Illustration of neurons (shown in red) in microfluidic chambers with cell bodies and proximal axons in the soma compartment on the right, and distal axons that have grown through the microfluidic grooves and extended into the axon compartment on the left. **B–F** Neurons isolated from p53-deficient mice were plated in microfluidic chambers and cisplatin was added to both soma and axon compartments (Global Cisplatin; 20 µM) to globally expose the whole cell to cisplatin for 96 h and stained for tubulin. The distal axons in the axon compartment are shown in **C** and the cell bodies and proximal axons are shown in **D**. Quantification of axon degeneration of **C** is shown in **E**; and quantification of axon degeneration in **D** is shown in **F**, individual data points and mean are depicted. All experiments were conducted with an *n* = 3; Paired *t*-test (two-tailed) (*p < 0.05). The scale bar in **C** and **D** depicts 100 μm.

To differentiate between these two possibilities, we examined the consequence of global cisplatin exposure to p53-deficient neurons and focused on the degeneration occurring in both the soma and axons. Importantly, since the microfluidic chamber model allows for the clearer differentiation of the proximal and distal axons from the soma, we conducted these experiments in microfluidic chambers where both compartments were exposed to cisplatin (Fig. 6A). We examined degeneration after 96 h of cisplatin exposure because at this point wild type neurons would have fully degenerated via apoptosis, but axons would not have completely degenerated from axonal only mtDNA damage. Our results showed that p53-deficiency conferred protection to the soma, but only partially (Fig. 6B). Interestingly, p53-deficiency also conferred protection to the proximal axons (Fig. 6D, F), while the distal axons were not protected and degenerated (Fig. 6C, E). These results indicate that p53 does not have a distinct function in mediating the degeneration in soma versus axons in response to global DNA damage. Rather, our observation that p53 deficiency protected the soma and proximal axons, albeit only partially, is consistent with p53 deficiency blocking the faster apoptotic pathway initiated from the nucleus but not the slower degenerative pathway initiated from mtDNA damage. Together, these results highlight the functional importance of p53 in mediating cell degeneration after nuclear but not mitochondrial DNA damage.

## Discussion

In this study we highlight the differential importance for p53 in governing the degenerative response to sustained nuclear versus mitochondrial DNA damage. While nuclear DNA damage is well known to activate an apoptotic cell death program, separating the distinct effects of nuclear versus mitochondrial DNA damage in cells has been experimentally challenging. Other than the few notable exceptions highlighted in the introduction, most studies investigating the molecular consequences of DNA damage in cells have been done in the context where the entire cell is exposed to DNA damage. In those situations, both nuclear and mitochondrial DNA are damaged, and the degeneration observed could be a combined effect of both nuclear and mitochondrial DNA damage pathways. The neuronal microfluidic model we utilize in this study allows for the selective induction of mtDNA damage in axons without damaging the nuclear DNA.

Our key observation is that selective mtDNA damage to the mitochondria in the axons is sufficient to activate a degenerative pathway that is spatially restricted to only the exposed regions. Surprisingly, this degenerative pathway was distinct, as it did not involve the major known axon degeneration pathways (e.g., apoptosis, axon pruning, or Wallerian degeneration). Interestingly, however, neurons deficient in p53 were partially protected from apoptosis after nuclear DNA damage but the lack of p53 did not protect distal axons from degenerating in response to mtDNA damage. Our findings here indicate that in response to DNA damage, two degenerative pathways are activated: apoptosis from nuclear DNA damage, and the degenerative response activated by mtDNA damage. Importantly, these two parallel degenerative pathways are normally not able to be differentiated in most model systems and likely occur in all contexts of global DNA damage.

Depending on the extent of DNA damage, cells can activate both repair and degenerative pathways. While we have some understanding of how this choice is made in the context of nuclear DNA damage, the pathways mediating the choice between mtDNA repair or degeneration are unknown. For nuclear DNA, p53 is important in determining the fate of the cell to repair damage or death via apoptosis in response to DNA damage^31^. Interestingly, recent reports have indicated that p53 also plays a role in mitochondrial maintenance^42,48^. For example, p53 has been show to both bind and transcriptionally regulate TFAM, the mitochondrial transcription factor important for mtDNA transcription and mtDNA maintenance^39,49^. p53 is also known to translocate to the mitochondria in contexts of stress^38,39^, and has been shown to be important for the maintenance of mtDNA^40–42^. For example, p53 is known to directly interact with the mtDNA repair and replication machinery including the mtDNA polymerase (POLG)^40^, human mitochondrial single-stranded binding protein (SSDP1)^50^, and the BER components 8-oxo-guanine glycosylase (OGG1) and apurinic endonuclease (APE)^41,51^. Our finding that p53-deficiency does not protect axons against mtDNA damage induced degeneration suggests that while p53 might play important roles in mtDNA replication and repair, it does not seem to play a similar role regulating survival versus degeneration like it does in response to nuclear DNA damage.

Our results with p53-deficient neurons globally exposed to DNA damage have revealed that distal axons are particularly vulnerable to mtDNA damage. One interesting question from our results is why might the proximal axons be more protected than distal axons in the experiment where p53-deficient neurons were globally exposed to cisplatin? One possible explanation is that as the proximal axons are closer to the protected soma, the large number of mitochondria in the soma are more readily available to replenish mitochondria in the proximal axons. However, this capability would decrease in the distal regions of the axons that are further away from the soma, leaving the distal axons most vulnerable to mtDNA damage.

The results we describe here also have broad relevance in the clinical context of chemotherapy-induced peripheral neuropathy (CIPN). CIPN is a progressive neurodegenerative condition in which the peripheral nervous system is damaged by chemotherapeutic drugs, such as cisplatin, used in cancer therapy. This condition can induce significant axon degeneration, leading to numerous neurological deficits, which are dose limiting factors for chemotherapy in cancer patients^52–55^. The increased vulnerability of peripheral axons to systemic chemotherapy is likely because of the large surface area of peripheral axons and their close association with the vasculature^56^. CIPN has been investigated in the context of paclitaxel exposure where it induces axon degeneration via disruption of microtubules^57^. The exact mechanism of this axon degeneration in cisplatin-induced CIPN is unclear but damage to mtDNA has been implicated^58^. Interestingly, distal axons have also been found to degenerate prior to proximal axons and cell body loss in CIPN^24,59^. Adult neurons are known to engage robust mechanisms to highly restrict apoptosis and protect their cell bodies^60,61^ while allowing selective axon degeneration and remodeling *via* axon pruning^30^. Thus, the apoptotic restriction in adult neurons is comparable to the outcome of our experiments with p53-deficient neurons, where the cell body is protected but distal axons degenerate from DNA damage.

DNA damaging chemotherapeutic drugs are generally assumed to exert their effect by damaging the nuclear DNA in rapidly dividing cells. However, it is notable that DNA damaging chemotherapeutic regiments also induce neuropathies (as discussed above) and cardiomyopathies^62–64^. These side effects are surprising because neurons and cardiomyocytes are post-mitotic cells that do not replicate their DNA and are thus not expected to be preferentially selected for damage by DNA damaging drugs. However, both neurons and cardiomyocytes are highly reliant upon active metabolisms and their mitochondria for optimal function. Our results draw distinctions between the nuclear, p53-dependent versus the mitochondrial, p53-independent consequences of chemotherapeutic drugs on cells.

## Materials and methods

### Primary sympathetic neuronal cultures

Primary sympathetic neurons were cultured as previously described^65^. Briefly, sympathetic neurons were dissected from the superior cervical ganglia (SCG) of postnatal day (P) 0-2 mice. Cells were plated directly into the microfluidic chamber device (described below) at a plating density of 10,000 cells per chamber and maintained for 5 days in vitro (DIV) in media containing NGF (AM50). Mice deficient for p53, Bax, Casp3, and Sarm1 are all from C57BL/6 backgrounds and wildtype littermates served as controls. All animal experiments were approved and conducted in compliance with the University of North Carolina at Chapel Hill Institutional Animal Care and Use Committee (IACUC).

### Fabrication and use of microfluidic devices

Microfluidic device master molds were generated using standard photolithographical procedures as previously described at the UNC CHANL facility^30^. Briefly, negative photoresist was utilized to pattern the chamber and microfluidic grooves using mylar chamber masks (Fineline Imaging). The microfluidic grooves were etched into a standard silicon wafer (Silicon Question Nation) with a groove depth of 3 μm with a groove width of 20 μm and with each groove being spaced 40 μm. The axon and soma compartments (750 μm length, 100 μm depth, 100 μm width for both soma and axon compartments) were added on either side of the etched grooves. To generate microfluidic devices, Sylgard 184 PDMS (Dow Corning) was prepared according to manufacturer protocol and poured into the mold form. The liquid mold was placed under vacuum at room temperature to remove air bubbles from the PDMS for 30 min then placed into a 95 °C oven to cure overnight. After curing, individual PDMS chambers were cut out and trimmed then soaked ddH2O overnight, sterilized in 70% EtOH, allowed to dry, mounted onto cleaned glass coverslips (Corning, CLS285025) coated overnight in 100 µg/ml poly-D-lysine (Sigma, P7886) and 1 µg/ml mouse laminin (Invitrogen, 23017-015), after mounting the chambers were then filled with poly-D-lysine and laminin solution and allowed to incubate for 72 h. Before neurons were plated into the soma compartment of the chambers, the chambers were washed 3 times with ddH2O and allowed to dry.

### Culture and treatment of primary neurons in microfluidic chambers

Neurons were plated in microfluidic chambers as described previously^22,30^ and maintained in AM50 until 5 DIV. To treat entire cells (both axon and soma chambers) with cisplatin, both the axon and soma compartment were washed three times with AM50 media containing cisplatin. To treat axons alone with cisplatin, only the axon compartment was washed with AM50 containing drug while the soma compartment is maintained in untreated AM50 media. A 10 μl volume differential was established between the soma and axon compartments and was reestablished every 12–20 h to maintain fluidic isolation of the two compartments^22^. For drug treatments, compounds were added to AM50 media at the following concentrations: 20 μM cisplatin (Sigma, P4394), 25 µM QVD-OPh (SM Biochemicals, SMPH001). All experiments were performed with three biological replicates.

### Axon degeneration quantification

Degeneration of axons was quantified from immunofluorescent images stained for tubulin taken in the microfluidic devices. The images are analyzed in ImageJ using a published and accepted axon degeneration analysis based on axon segment continuity^66^. Data was quantified from eight biological replicates.

### Immunofluorescence

Immunofluorescence staining was carried out as previously described^30^. Briefly, the two compartments are stained by added solutions to the top chamber reservoirs and allowing solutions to flow through into the chamber areas. The following primary antibodies were used for staining: alpha-tubulin (Sigma, T9026), Tom20 (Santa Cruz, sc-11415), cyt *c* (BD Scientific, 556432), ɣH2AX (Cell Signaling, 9718 s). Nuclei were labeled with Hoechst 33258 (ThermoFisher, H3569). The following mitochondrial markers used are: Mitotracker Green FM (Invitrogen, M7514, used at 100 nM), CM-H_2_DCFDA (Invitrogen, C6827, used at 10 µM), TMRE (Invitrogen, T669, used at 50 nM). Quantification of cyt *c* release was conducted using Coloc 2 in ImageJ for colocalization using the Pearson correlation coefficient on cyt *c* and Tom20 stained images.

### Western analysis

Whole brain lysate from wildtype and knock-out animals was analyzed by Western blot for validation of gene knock-out using the following antibodies: Bax N20 (Santa Cruz, SC-493, 1:1000); Caspase-3 (BD Biosciences, 559565, 1:1000); Sarm1 (Abcam, ab226930, 1:1000); beta-Actin (Sigma, A5316, 1:1000).

### mtDNA damage analysis

mtDNA was isolated by collecting axonal material from the axon compartment of treated and untreated axons using a DNeasy Blood & Tissue kit (Qiagen, 69504). The mtDNA was then amplified using primers specific to short (117 bp) and long (10 kb) amplicons of murine mtDNA as previously described^25,26^. Briefly, the assay compares the amplification of a long versus short segment of mtDNA to generate an amplification ratio; when mtDNA is damaged the ratio of long:short amplification is reduced as DNA damage that blocks Taq polymerase processivity occurs with a higher frequency on longer DNA segments than on shorter fragments. The 117 bp short region used the following for amplification: forward primer: 5′-CCC AGC TAC TAC CAT CAT TCA AGT-3′, reverse primer: 5′-GAT GGT TTG GGA GAT TGG TTG ATG T-3′. The 10 kb long region used the following for amplification: forward primer: 5′-GCC AGC CTG ACC CAT AGC CAT AAT AT-3′, reverse primer: 5′-GAG AGA TTT TAT GGG TGT AAT GCG G-3′. PCR was conducted using Platinum Blue TAQ (Invitrogen, 12580015) and the conditions for amplification were followed as previously described^26^. The amplified mtDNA products were measured using a Picogreen DNA amplification assay as previously described using a Thermo Fluoroskan Ascent FL fluorescent plate reader^25^. Resulting values of the short and long mtDNA amplification fragments were then ratiometrically compared as previously reported to quantify the amount of mtDNA damage in a sample^25,26^. Experiments were conducted in biological triplicate.

### Image acquisition and processing

Images were acquired on a DMI6000 inverted fluorescent microscope (Leica) using an ORCA-ER B/W CCD camera (Hamamatsu; native resolution 1344 × 1024 pixels) through Metamorph software (Molecular Devices, version 7.6) or on a DMI8 inverted fluorescent microscope (Leica; native resolution 2048 × 2048 pixels) using a Leica DFC9000 sCMOS camera through LASX software (Leica). Axons and soma were imaged using ×10 and ×20 air lenses. Images of axons showing CM-H_2_DCFDA, cyt *c*, and TMRE were imaged using ×63 oil lenses.

## Supplementary information

Supplemental Figure Legend

Supplemental Figure 1

Supplemental Figure 2
