# Supplementary material for: p53 is required for nuclear but not mitochondrial DNA damage-induced degeneration
Source: Cell Death Dis. 2021 Jan 20;12(1):104. doi: 10.1038/s41419-020-03373-1 (PMC7817838; doi:10.1038/s41419-020-03373-1)
Supplement: Supplementary file 1 — Supplemental Figure Legend [file 41419_2020_3373_MOESM1_ESM.docx]

**Supplemental Figures**

**Supplemental Fig. 1. Cisplatin-induced mtDNA damage in axons elevates ROS levels but not loss of mitochondrial membrane potential.** Neurons were cultured in microfluidic chambers and had only their axons exposed to cisplatin (20 μM) at t=0 hours and were monitored for degeneration by microscopy every 24 hours for 96 hours total. As a positive control, neurons were also subjected to NGF deprivation (in the presence of QVD-OPH) for 48 hours. **(A)** ROS levels were assessed with the addition of the ROS-sensitive dye CM-H_2_DCFDA (10 uM) at each timepoint. Shown are representative images of treated axons with phase imaging (top panels) and ROS (bottom panels). **(B)** Mitochondrial membrane potential was examined with the addition of TMRE (50 nM) at each timepoint. Mitochondria were also labeled with Mitotracker Green (100 nM). Shown are representative images of treated axons with Mitotracker Green (top panels) and TMRE (bottom panels).

**Supplemental Fig. 2. Western blots validating various knock-out mice.** Whole brain lysates from wildtype and mice deleted for Bax **(A)**, Caspase-3 **(B)**, and Sarm1 **(C)** subjected to Western analysis with antibodies to Bax, Caspase-3, or Sarm1, respectively. Actin is shown as a loading control.
