# Supplementary figures and images for: p53 is required for nuclear but not mitochondrial DNA damage-induced degeneration
Source: Cell Death Dis. 2021 Jan 20;12(1):104. doi: 10.1038/s41419-020-03373-1 (PMC7817838; doi:10.1038/s41419-020-03373-1)

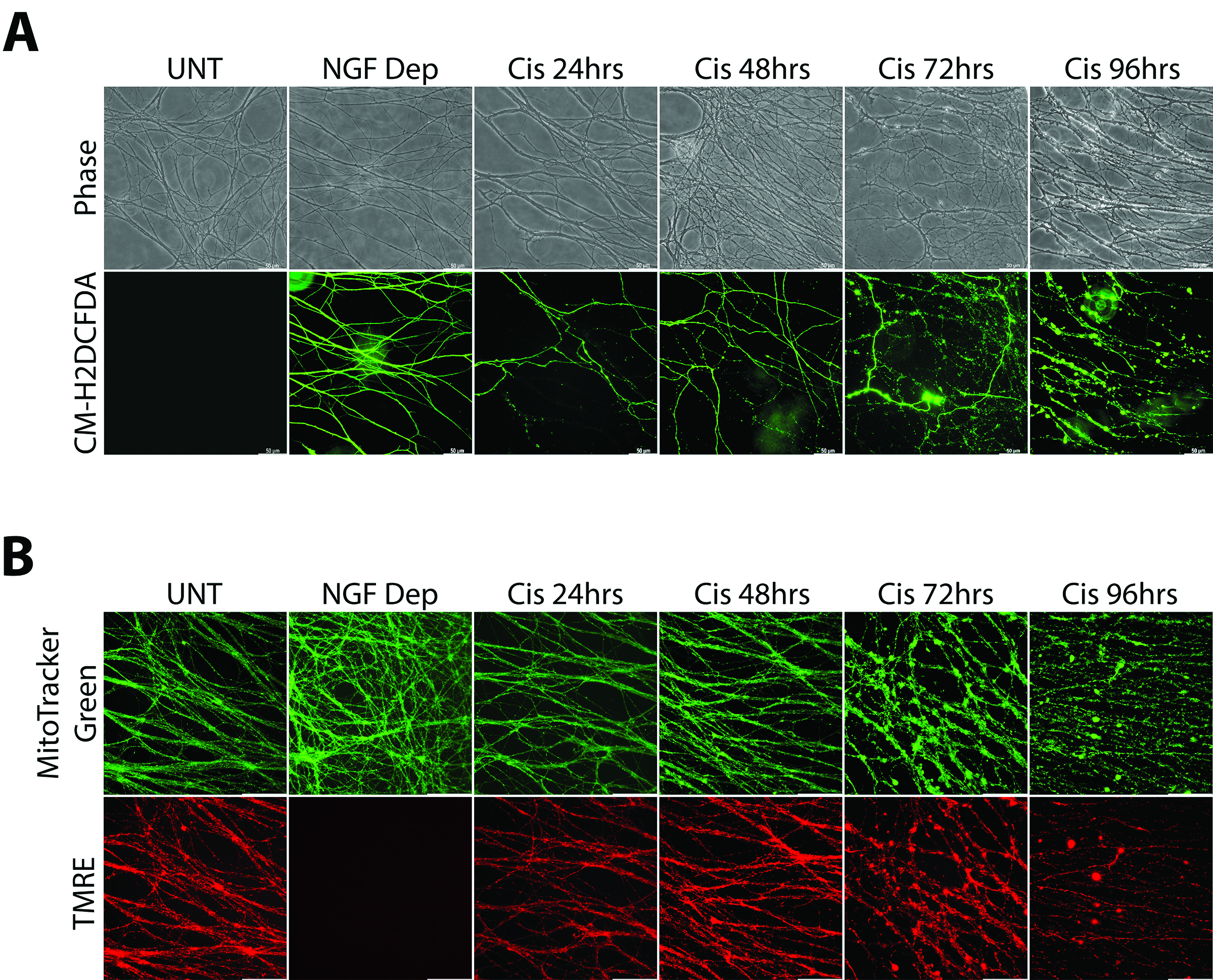

Supplement: Supplementary file 2 — Supplemental Figure 1 [file 41419_2020_3373_MOESM2_ESM.tif]

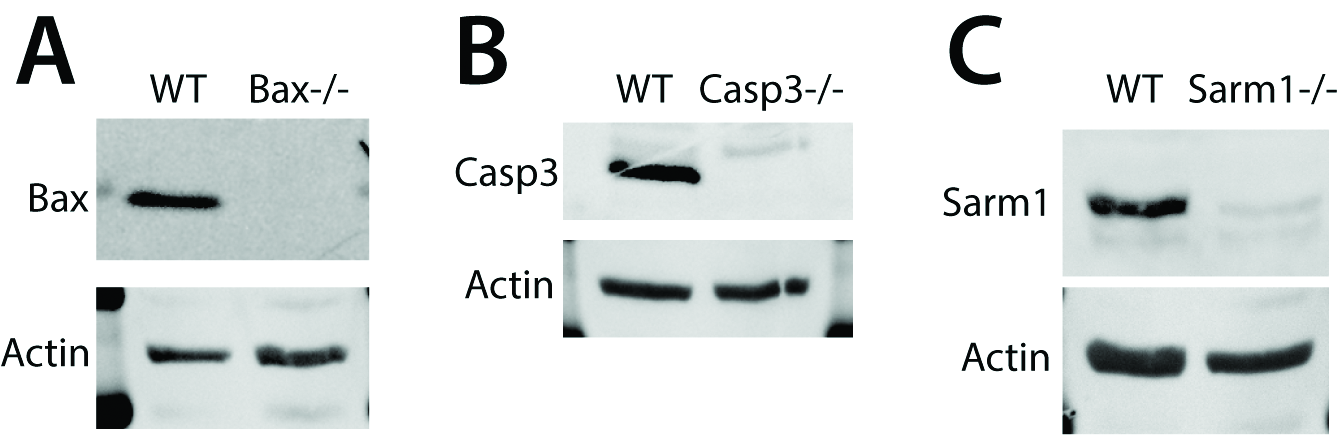

Supplement: Supplementary file 3 — Supplemental Figure 2 [file 41419_2020_3373_MOESM3_ESM.tif]
